# Motor imagery and action observation for predictive control in developmental coordination disorder

**DOI:** 10.1111/dmcn.14612

**Published:** 2020-07-31

**Authors:** Bert Steenbergen, Hilde Krajenbrink, Jessica Lust, Peter Wilson

**Affiliations:** ^1^ Behavioural Science Institute (BSI) Radboud University Nijmegen the Netherlands; ^2^ Centre for Disability and Development Research (CeDDR) School of Behavioural and Health Sciences Australian Catholic University Melbourne VIC Australia

## Abstract

In 2019, international clinical practice recommendations on the definition, diagnosis, assessment, intervention, and psychosocial aspects of developmental coordination disorder (DCD) were published. Informing our understanding of mechanisms, recent systematic reviews have shown that children with DCD have difficulties with the predictive control of movements, including aspects of motor planning, which is expressed as the internal modeling deficit hypothesis. This motor control deficit is most evident when the spatial and temporal demands of a task increase. An increasing number of empirical studies suggest that motor planning problems can be remediated through training based on one or a combination of motor imagery and action observation. In this review, we show evidence of motor planning problems in children with DCD and show that task demands or complexity affects its appearance. Implications of these findings are treatments based on motor imagery and action observation to remediate motor planning issues. The article concludes with recommendations for future research.

## Abstract 2

This article's abstract has been translated into Spanish and Portuguese.

Follow the links from the abstract to view the translations.

## Imaginería motora y observación de la acción para el control predictivo en el trastorno de coordinación del desarrollo

En 2019, se publicaron recomendaciones de práctica clínica internacional sobre la definición, el diagnóstico, la evaluación, la intervención y los aspectos psicosociales del trastorno de coordinación del desarrollo (TCD). Para informar nuestra comprensión de los mecanismos, revisiones sistemáticas recientes han demostrado que los niños con TCD tienen dificultades con el control predictivo de los movimientos, incluidos los aspectos de la planificación motora, que se expresa como la hipótesis del déficit de modelado interno. Este déficit de control motor es más evidente cuando aumentan las demandas espaciales y temporales de una tarea. Un número creciente de estudios empíricos sugiere que los problemas de planificación motora pueden remediarse mediante el entrenamiento basado en una o una combinación de imaginería motora y observación de la acción. En esta revisión, mostramos evidencia de problemas de planificación motora en niños con TCD y mostramos que las demandas o la complejidad de la tarea afectan su apariencia. Las implicaciones de estos hallazgos son tratamientos basados en imaginería motora y observación de acciones para remediar problemas de planificación motora. El artículo concluye con recomendaciones para futuras investigaciones.

## Imagética motora e observação da ação para controle preditivo em transtorno do desenvolvimento da coordenação

Em 2019, recomendações internacionais de prática clínica sobre a definição, diagnóstico, avaliação, intervenção, e aspectos psicossociais do transtorno do desenvolvimento da coordenação (TDC) foram publicadas. Informando nossa compreensão sobre mecanismos, revisões sistemáticas recentes têm motrado que crianças com TDC têm dificuldades com o controle preditivo dos movimentos, incluindo aspectos do planejamento motor, o qual se expressa como a hipótese do déficit de modelo interno. Este déficit do controle motor é mais evidente quando as demandas especiais e temporais da tarefa aumentam. Um número crescente de estudos empíricos sugere que os problemas de planejamento motor podem ser remediados por meio de treinamento baseado em uma combinação de imagética motora e observação da ação. Nesta revisão, mostramos evidências de problemas de planejamento motor em crianças com TDC e mostramos que as demandas ou complexidade da tarefa afetam seu aparecimento. Implicações destes achados são tratamentos baseados na imagética motora e observação da ação para remediar os problemas de planejamento motor. O artigo conclui com recomendações para pesquisas futuras.

**Abbreviation**
DCD: Developmental coordination disorder

**What this paper adds**

- Task complexity is an important factor for the appearance of planning problems in developmental coordination disorder (DCD).
- Evidence for motor imagery and action observation intervention to remediate motor problems in DCD is mounting.
- Growing evidence supports the use of motor imagery and action observation to remediate motor problems in DCD.

The latest edition of the international clinical recommendations of developmental coordination disorder (DCD) were published in 2019.^1^ Compared with earlier editions, there has been a noticeable shift in the conceptualization of DCD as a pure motor syndrome to a disorder characterized by more complex motor–cognitive issues. Research has shown deficits across different aspects of motor control and motor learning, as well as cognitive control (executive function). Accordingly, the 2019 guidelines recommended that both motor and non‐motor aspects of functioning for an individual child with DCD be considered when setting up an intervention (recommendation 21). In this paper we first provide an overview of current research on motor planning in children with DCD, noting in particular the effect of task demands or complexity on the expression of planning deficits. In the second part we discuss recent evidence on the use of motor imagery and action observation (action observation) for remediation of these motor problems.

## MOTOR PLANNING AND TASK COMPLEXITY

Sound motor planning is important for a wide variety of daily activities, such as picking up and drinking from a cup, opening a door, or navigating through a crowded room. While many such actions have no serious consequences when planning goes awry, other situations can be far more hazardous, such as crossing a busy street.^2^ This situation poses a complex planning problem, with cars approaching from left and right at different closing speeds, and in countries like the Netherlands, cyclists crossing unpredictably. In these complex environments it is of utmost importance that planning is performed with great precision and well before the action is initiated. Put simply, proper (motor) planning is essential for the safe and efficient performance of many activities in daily life.

In a recent systematic review of experimental research on mechanisms of DCD, Wilson et al.^3^ identified sustained growth of work, with a total of 106 studies published between September 2011 and September 2017. On the strength of good methodological quality, the review clearly showed that the expression of motor control deficits was dependent on the nature of the tasks and their complexity. That is, motor problems were most evident under conditions of high task complexity (e.g. dual‐tasks, tasks demanding more precision, tasks performed under tight temporal constraints and/or under restricted visual conditions). Moreover, there was converging evidence to support deficits in the predictive control of movements, as poor anticipatory planning was a common denominator in the many studies.

Motor control theories state that when an action is planned, the motor parameters related to the action such as trajectory, velocity, force, and required precision are represented as internal or feedforward models.^4^, ^5^ Internal models contribute to smooth, efficient, and accurate motor performance, reducing the necessity for the motor system to rely on slower forms of feedback‐based control. According to the internal modeling deficit hypothesis, children with DCD have reduced capacity to use internal modeling as a means of motor control.^6^, ^7^ This has a severe impact on motor learning, which is consistent with the movement profile of children with DCD who often show slow, laboured, and effortful motor performance, a characteristic of more reliance on slow forms of feedback‐based control. In addition, the internal modeling deficit hypothesis provides a theoretical account for the pattern of deficits in predictive control that have been observed repeatedly in children with DCD.^3^ Since its first iteration in 2001, this hypothesis has garnered support from a large body of research in children with DCD^3^, ^8^ as well as in cerebral palsy.^9^

Studies have also shown consistent effects of task demands and complexity on the appearance of motor planning problems in DCD. Importantly, they indicate a broad cluster of deficits in the anticipatory control of reaching movements, linked to the internal modeling process.^10^ Studies of motor planning in DCD have relied mainly on paradigms that ask children to plan for end‐state comfort,^11^ for example, grasping an upturned glass using an awkward pronated posture so as to finish with the glass turned upright in a comfortable end position. End‐state comfort planning is needed to attenuate the signal‐to‐noise ratio of position errors at the end of the task, thereby allowing greater precision to be exerted.^12^ There is evidence, however, that striving for end‐state comfort is not likely to be the only strategy for motor planning in children with poor motor control.^13^ Changes in task demands may necessitate the use of other strategies as well. For example, using the octagon task, Bhoyroo et al.^14^ showed that children with DCD adopted both a planning strategy based on end‐state comfort as well as planning based on a minimal rotation strategy.

The recent meta‐analysis by Bhoyroo et al.^15^ indicated effects of task complexity on planning problems in DCD. These children were able to plan for comfortable end states on tasks of a low and moderate complexity level, but showed impairments on tasks of high complexity compared with their typically developing peers. Manipulation of complexity on these various tasks was categorized in to four dimensions: (1) the number of grip choices, (2) the level of end‐point precision, (3) the number of action steps, and (4) the degree of biomechanical rotation. The authors showed that the degree to which motor planning problems were observed was related to the complexity of the task. For example, in the octagon task, participants perform sequences of one, two, or three movements by rotating an octagon towards one, two, or three colour(s) respectively. It was shown that sequence length had a direct effect on planning success. That is, the longer the sequence, the more disadvantaged children with DCD were at the task. Collectively, an increasing number of studies support the effect of task demands/complexity on the appearance of the planning deficit.^3^, ^15^, ^16^ An important avenue for future studies is to systematically examine the complexity of the task and its effects on the grip selection and movement strategy used. Manipulations of each of the four dimensions of complexity identified by Bhoyroo et al.^15^ have only been studied in isolation. A more systematic study of the richness of motor planning in children with DCD would involve a parametric analysis of these dimensions.

To complement group‐based research, an idiographic approach is also suggested, consistent with ecological models of motor behaviour.^17^ Future studies should aim to identify the specific parameters of a motor task that either improve or impede the performance of an individual child (see also the ecological task analysis by Davis and Burton^18^). This approach has implications for task‐oriented approaches to training, and personalized treatment programmes for a given child. The issue of how motor planning can be remediated is addressed in the next section. We propose two possible training modes, motor imagery and action observation, that are based on its known neural overlap with internal modeling. It is beyond the scope of this paper to elaborately discuss the neurological aspects of these training modes, but for an excellent review, see Vogt et al.^19^

## THE USE OF MOTOR IMAGERY AND ACTION OBSERVATION FOR REMEDIATION OF MOTOR PLANNING PROBLEMS

We have shown that an increasing number of studies provide evidence for compromised motor planning in DCD, and the effect of task demands and complexity on its expression. An important next step is to translate this knowledge into clinical practice recommendations by development of feasible and effective intervention programmes for children with DCD. As stated above, motor planning problems in DCD have been interpreted within the internal modeling deficit hypothesis.^3^ This hypothesis provides a good entry point for training programmes, under the assumption that strategies and techniques that support the internal modeling process may improve motor learning. Our working hypothesis is that children with predictive control issues may need additional information to build an adequate ‘internal image’ of the action they are about to learn. In recent years, two techniques have been put forward that may be used for DCD to scaffold motor control and learning: motor imagery training and action observation training. Both motor imagery and action observation can be regarded as forms of motor simulation, targeting the motor system, while not focusing on overt movement as the main treatment modality. Motor imagery entails an imagined action, while action observation involves the observation of someone else performing an action. Importantly, motor imagery and action observation involve brain areas that are also involved in motor planning and execution,^20^ and they share neural networks that have been associated with the internal modeling process.^3^ This feature of motor imagery and action observation provides a neurophysiologically feasible locus for training motor planning and performance, as evidenced in other reviews.^18^

Eaves et al.^21^ have formulated a very useful conceptual framework for the combined use of motor imagery and action observation. Here, the participant is instructed to imagine the kinesthetic and physiological sensations associated with an action and to subsequently synchronize these with a congruent action, modeled on a video clip, for example. The empirical evidence of this approach has been shown both at a behavioural and neurophysiological level, initially with adults, but it also seems to be promising with children.^21^ That is, in line with the model, the combined use of motor imagery and action observation was a more effective tool for impacting motor skills in children with varying motor abilities compared with motor imagery and action observation in isolation. Recent work by Scott et al.^22^ and Marshall et al.^23^ provided further empirical support for the improved efficacy of the combined use of motor imagery and action observation over and above the isolated use of either motor imagery or action observation. Marshallet al.,^23^ for example, tested the combined use of motor imagery and action observation to examine its effect on the kinematics of eye–hand coordination during a visuomotor rotation task. The results showed that motor performance improved in children with DCD, which, according to the authors, provides further support for the internal modeling deficit hypothesis. Thus, studies have started to emerge showing the benefit of combined action observation and motor imagery interventions to alleviate motor problems in children with DCD.

Protocols for the systematic use of these techniques have been proposed^24^, ^25^ and both have been tested in children with DCD^26^, ^27^ and cerebral palsy.^28^ In general, these intervention studies have been promising. For example, Wilson et al.^29^ used 4 weeks of motor imagery training (that also involved action observation) in a group of children with DCD and compared it with traditional perceptual‐motor training. The results of this first study showed improvements on Movement Assessment Battery for Children, First Edition scores for the motor imagery training group that were comparable to those observed in the perceptual‐motor training group. Importantly, these results were replicated in a group of children with more severe DCD, using the same set‐up.^27^ Also Adams et al.^26^ showed preliminary evidence of improved performance on the Movement Assessment Battery for Children, Second Edition and perceived benefits of training for new skill learning (reported by parents and children). The collective results of these studies in combination with those mentioned above provides a strong case for the efficacy of combined motor imagery and action observation training in DCD and warrant more systematic testing to identify the most active ingredients. Taken together, use of motor imagery and action observation, either in isolation or combined, are potentially important rehabilitation tools for children with DCD.

There are several issues that future studies on motor imagery/action observation interventions need to address. First, if these techniques are to be used in children, it is imperative to assess if the child understands the very idea of imagined action and, if so, has sufficient cognitive control to implement it. Given that issues in both motor control and cognition are common in DCD, many (particularly younger) children may find these cognitive techniques difficult without adequate support. In a recent electrophysiological study, Lust et al.^30^ showed that children with DCD have problems with the correct coupling of movement goals and means during observation motor learning. In addition, a review on the development of motor imagery in typically developing children by Spruijt et al.^31^ showed that performance on imagery tasks develops steadily between 5 and 10 years of age, while the exact age of onset of motor imagery may vary with the complexity of the imagined action. Therefore, it is advisable to first use a simple test of motor imagery ability^32^ for the individual child, before training techniques are implemented so as to ascertain appropriate starting points for instruction.

Second, the exact intensity and programming of the training is important to consider. Generally speaking, high intensity is more effective, but it is important to consider the attentional capacity of children with DCD, which is commonly reduced relative to typically developing peers. In the motor imagery‐replication study by Wilson et al.,^27^ individual training was conducted over 5 weeks, with one 60 minute session per week. It is likely that shorter and more frequent sessions may be more effective, but this remains a matter of empirical testing. In this respect it is important to take recommendation 21 of the international clinical recommendations of DCD into account: ‘both motor and non‐motor aspects of functioning of a specific child with DCD are to be considered when setting up an intervention’.^1^ Problems in executive function encompass aspects of selective and sustained attention, which are also at the basis of interventions using motor imagery or action observation. It is possible that engaging forms of video modeling can attenuate the impact of these issues in executive function, but there remains a need to develop more specific techniques to scaffold cognition.^29^

Third, the type of training activity deserves careful attention. In the case of action observation, Buccino et al.^28^, ^33^ used movie clips of 15 activities of daily living, but did not report data on the efficacy of individual clips. This information is nonetheless important to refine and inform future clinical training and to improve its efficacy. From a child‐centred perspective, it is advisable that children play a role in selecting those skills that are a target of intervention. Nothing will breed persistence and motivation more than self‐selected goals and tasks.

In sum, basic research has shown the feasibility of motor imagery and action observation training to enhance motor performance in children with DCD. While efficacy has been shown in several pilot studies, the field has now moved to more systematic studies on the efficacy of motor imagery and action observation, and their combined use. The challenge now is to identify specific task parameters that can be targeted in training regimes that can be shown to transfer training effects from simple motor skills to more elaborate activities of daily living and performance.

## References

[1] Blank R , Barnett AL , Cairney J , et al. International clinical practice recommendations on the definition, diagnosis, assessment, intervention, and psychosocial aspects of developmental coordination disorder. Dev Med Child Neurol 2019; 61: 242–85. 30671947 10.1111/dmcn.14132 PMC6850610

[2] Purcell C , Wilmut K , Wann JP . The use of visually guided behaviour in children with developmental coordination disorder (DCD) when crossing a virtual road. Hum Mov Sci 2017; 53: 37–44. 27939726 10.1016/j.humov.2016.11.007

[3] Wilson PH , Smits‐Engelsman B , Caeyenberghs K , et al. Cognitive and neuroimaging findings in developmental coordination disorder: New insights from a systematic review of recent research. Dev Med Child Neurol 2017; 59: 1117–29. 28872667 10.1111/dmcn.13530

[4] Wolpert DM . Computational approaches to motor control. Trends Cogn Sci 1997; 1: 209–16. 21223909 10.1016/S1364-6613(97)01070-X

[5] Kawato M . Internal models for motor control and trajectory planning. Curr Opin Neurobiol 1999; 9: 718–27. 10607637 10.1016/s0959-4388(99)00028-8

[6] Wilson PH , Maruff P , Ives S , Currie J . Abnormalities of motor and praxis imagery in children with DCD. Hum Move Sci 2001; 20: 135–59. 10.1016/s0167-9457(01)00032-x 11471394

[7] Katschmarsky S , Cairney S , Maruff P , Wilson PH , Currie J . The ability to execute saccades on the basis of efference copy: Impairments in double‐step saccade performance in children with developmental co‐ordination disorder. Exp Brain Res 2001; 136: 73–8. 11204415 10.1007/s002210000535

[8] Adams ILJ , Lust JM , Wilson PH , Steenbergen B . Compromised motor control in children with DCD: A deficit in the internal model?—A systematic review. Neurosci Biobehav Rev 2014; 47: 225–44. 25193246 10.1016/j.neubiorev.2014.08.011

[9] Steenbergen B , Jongbloed‐Pereboom M , Spruijt S , Gordon AM . Impaired motor planning and motor imagery in children with unilateral spastic cerebral palsy: Challenges for the future of pediatric rehabilitation. Dev Med Child Neurol 2013; 55: 43–6. 24237279 10.1111/dmcn.12306

[10] Adams ILJ , Lust JM , Wilson PH , Steenbergen B . Testing predictive control of movement in children with developmental coordination disorder using converging operations. Br J Psychol 2017; 108: 73–90. 26861617 10.1111/bjop.12183

[11] Rosenbaum DA , Vaughan J , Barnes HJ , Jorgensen MJ . Time course of movement planning: Selection of handgrips for object manipulation. J ExpPsychol: Learn Mem Cogn 1992; 18: 1058–73. 10.1037//0278-7393.18.5.1058 1402710

[12] Harris CM , Wolpert DM . Signal‐dependent noise determines motor planning. Nature 1998; 394: 780–4. 9723616 10.1038/29528

[13] Steenbergen B , Hulstijn W , Dortmans S . Constraints on grip selection in cerebral palsy. Exp Brain Res 2000; 134: 385–97. 11045363 10.1007/s002210000458

[14] Bhoyroo R , Hands B , Wilmut K , Hyde C , Wigley A . Motor planning with and without motor imagery in children with developmental coordination disorder. Acta Psychol 2019; 199: 102902. 10.1016/j.actpsy.2019.102902 31404744

[15] Bhoyroo R , Hands B , Steenbergen B , Wigley CA . Examining complexity in grip selection tasks and consequent effects on planning for end‐state‐comfort in children with developmental coordination disorder: A systematic review and meta‐analysis. Child Neuropsychol 2019; 26: 534–59. 31766934 10.1080/09297049.2019.1695768

[16] Adams ILJ , Ferguson GD , Lust JM , Steenbergen B , Smits‐Engelsman BCM . Action planning and position sense in children with developmental coordination disorder. Hum Move Sci 2016; 46: 196–208. 10.1016/j.humov.2016.01.006 26796420

[17] Adolph KE . An ecological approach to learning in (not and) development. HumDev 2019; 63: 180–201. 10.1159/000503823 PMC8048368 33867566

[18] Davis WE , Burton AW . Ecological task analysis: Translating movement behavior theory into practice. A Adapt Phys Activ Q 1991; 8: 154–77.

[19] Vogt S , Di Rienzo F , Collet C , Collins A , Guillot A . Multiple roles of motor imagery during action observation. Front Hum Neurosci 2013; 7: 807. 24324428 10.3389/fnhum.2013.00807 PMC3839009

[20] Grèzes J , Decety J . Functional anatomy of execution, mental simulation, observation, and verb generation of actions: A meta‐analysis. Hum Brain Map 2001; 12: 1–19. 10.1002/1097-0193(200101)12:1<1::AID-HBM10>3.0.CO;2-V PMC6872039 11198101

[21] Eaves DL , Riach M , Holmes PS , Wright DJ . Motor imagery during action observation: A brief review of evidence, theory and future research opportunities. Front Neurosci 2016; 10: 514. 27917103 10.3389/fnins.2016.00514 PMC5116576

[22] Scott MW , Emerson JR , Dixon J , Tayler MA , Eaves DL . Motor imagery during action observation enhances automatic imitation in children with and without developmental coordination disorder. J Exp Child Psychol 2019; 183: 242–60. 30921604 10.1016/j.jecp.2019.03.001

[23] Marshall B , Wright DJ , Holmes PS , Williams J , Wood G . Combined action observation and motor imagery facilitates visuomotor adaptation in children with developmental coordination disorder. Res Dev Disabil 2020; 98: 103570. 31918039 10.1016/j.ridd.2019.103570

[24] Adams ILJ , Steenbergen B , Lust JM , Smits‐Engelsman BCM . Motor imagery training for children with developmental coordination disorder – Study protocol for a randomized controlled trial. BMC Neurol 2016; 16: 5. 26758026 10.1186/s12883-016-0530-6 PMC4710999

[25] Buccino G . Action observation treatment: A novel tool in neurorehabilitation. Philos Trans R Soc Lond B Biol Sci 2014; 369: 20130185. 24778380 10.1098/rstb.2013.0185 PMC4006186

[26] Wilson PH , Adams ILJ , Caeyenberghs K , Thomas P , Smits‐Engelsman B , Steenbergen B . Motor imagery training enhances motor skill in children with DCD: A replication study. Res Dev Disabil 2016; 57: 54–62. 27388492 10.1016/j.ridd.2016.06.014

[27] Adams ILJ , Smits‐Engelsman B , Lust JM , Wilson PH , Steenbergen B . Feasibility of motor imagery training for children with developmental coordination disorder – a pilot study. Front Psychol 2017; 8: 1271. 28798707 10.3389/fpsyg.2017.01271 PMC5526967

[28] Buccino G , Molinaro A , Ambrosi C , et al. Action observation treatment improves upper limb motor functions in children with cerebral palsy: A combined clinical and brain imaging study. Neural Plast 2018; 2018: 11. 10.1155/2018/4843985 PMC6079352 30123250

[29] Wilson PH , Thomas PR , Maruff P . Motor imagery training ameliorates motor clumsiness in children. J Child Neurol 2002; 17: 491–8. 12269727 10.1177/088307380201700704

[30] Lust JM , van Schie HT , Wilson PH , van der Helden J , Pelzer B , Steenbergen B . Activation of mirror neuron regions is altered in developmental coordination disorder (DCD) ‐ Neurophysiological evidence using an action observation paradigm. Front Hum Neurosci 2019; 13: 232. 31354451 10.3389/fnhum.2019.00232 PMC6637752

[31] Spruijt S , van der Kamp J , Steenbergen B . Current insights in the development of children’s motor imagery ability. Front Psychol 2015; 6: 787. 26113832 10.3389/fpsyg.2015.00787 PMC4461854

[32] Spruijt S , Jouen F , Molina M , Kudlinski C , Guilbert J , Steenbergen B . Assessment of motor imagery in cerebral palsy via mental chronometry: The case of walking. Res Dev Disabil 2013; 34: 4154–60. 24076980 10.1016/j.ridd.2013.08.044

[33] Buccino G , Arisi D , Gough P , et al. Improving upper limb motor functions through action observation treatment: A pilot study in children with cerebral palsy. Dev Med Child Neurol 2012; 54: 822–8. 22765352 10.1111/j.1469-8749.2012.04334.x

